# Expression of ventral telencephalon transcription factors ASCL1 and DLX2 in the early fetal human cerebral cortex

**DOI:** 10.1111/joa.12971

**Published:** 2019-03-12

**Authors:** Ayman Alzu'bi, Gavin J. Clowry

**Affiliations:** ^1^ The institute of Neuroscience Newcastle University Newcastle upon Tyne UK; ^2^ The Institute of Genetic Medicine Newcastle University Newcastle upon Tyne UK; ^3^ The Department of Basic Medical Sciences Yarmouk University Irbid Jordan

**Keywords:** cortical inhibitory interneurons, development, dorsal telencephalon, ganglionic eminences

## Abstract

In rodent ventral telencephalon, diffusible morphogens induce expression of the proneural transcription factor ASCL1, which in turn induces expression of the transcription factor DLX2 that controls differentiation of cortical interneuron precursors and their tangential migration to the cerebral cortex. RNAseq analysis of human fetal samples of dorsal telencephalon revealed consistently high cortical expression of *ASCL1* and increasing expression of *DLX2* between 7.5 and 17 post‐conceptional weeks (PCW). We explored whether cortical expression of these genes represented a population of intracortically derived interneuron precursors. Immunohistochemistry revealed an ASCL1^+^/DLX2^+^ population of progenitor cells in the human ganglionic eminences between 6.5 and 12 PCW, but in the cortex there also existed a population of ASCL1^+^/DLX2^–^ progenitors in the subventricular zone (SVZ) that largely co‐expressed cortical markers PAX6 or TBR2, although a few ASCL1^+^/PAX6^–^ progenitors were observed in the ventricular zone (VZ) and ASCL1^+^ cells expressing the interneuron marker GAD67 were present in the SVZ. Although rare in the VZ, DLX2^+^ cells progressively increased in number between 8 and 12 PCW across the cortical wall and the majority co‐expressed LHX6 and originated either in the MGE, migrating to the lateral cortex, or from the septum, populating the medial wall. A minority co‐expressed COUP‐TFII, which identifies cells from the caudal ganglionic eminence (CGE). By 19 PCW, a significant increase in expression of DLX2 and ASCL1 was observed in the cortical VZ with a small proportion expressing both proteins. The DLX2^+^ cells did not co‐express a cell division marker, so were not progenitors. The majority of DLX2^+^ cells throughout the cortical plate expressed COUP‐TFII rather than LHX6^+^. As the VZ declined as a proliferative zone it appeared to be re‐defined as a migration pathway for COUP‐TFII^+^/DLX2^+^ interneurons from CGE to cortex. Therefore, in developing human cortex, ASCL1 expression predominantly marks a population of intermediate progenitors giving rise to glutamatergic neurons. DLX2 expression predominantly defines post‐mitotic interneuron precursors.

## Introduction

The ventral and dorsal identities of the telencephalon are chiefly regulated by antagonistic interplay between the secreted signalling protein SHH and the dorsalising zinc‐finger transcription factor GLI3. These two signals establish the early pallial/subpallial (PSB) boundary between dorsal and ventral telencephalon (Ericson et al. 1995; Rallu et al. 2002; Campbell, 2003; Hébert & Fishell, 2008) with ventralising SHH secreted from the ventral midline of the diencephalon (Ericson et al. 1995) and the globus pallidus later in development (Memi et al. 2018), and GLI3 expressed throughout the dorsal telencephalon and repressing the SHH signal. Loss of GLI3 function leads to ectopic expression of ventral telencephalon markers in the cortex (Rallu et al. 2002). SHH induces the expression of NKX2.1, without which the medial ganglionic eminence (MGE) fails to develop (Pabst et al. 2000; Rallu et al. 2002). However, the expression of other transcription factors that are expressed in both the MGE and LGE (including *Dlx* genes) is conserved in these mutants, suggesting that SHH is only required for patterning the MGE (Rallu et al. 2002) and so SHH‐independent signalling pathways may also act during development of the ventral telencephalon.

ASCL1 (also known as MASH1) is a proneural transcription factor that is highly expressed in the proliferative zones throughout the rodent ventral telencephalon (Fode et al. 2000). It promotes expression of the Notch ligand Delta 1, which mediates lateral inhibition through the Notch signalling pathway to prevent precocious differentiation of neural progenitors (Casarosa et al. 1999; Horton et al. 1999; Yun et al. 2002) and also promotes specification of ventral telencephalic identity by transactivating expression of *Dlx* and *Gad* genes (Poitras et al. 2007; Castro et al. 2011). DLX1/2 are closely related transcription factors which suppress ASCL1 expression and promote differentiation and migration of GABAergic neuron precursors, including cortical interneuron precursors, which principally derive from the MGE and the caudal ganglionic eminence (CGE; Anderson et al. 1997; Cobos et al. 2005; Long et al. 2009). In the mouse, there is also low expression of ASCL1 in the dorsal telencephalon (Britz et al. 2006), where it plays a different role by promoting maturation of progenitors and their transition from the VZ to the SVZ (Britz et al. 2006).

Interpretation of the expression of ASCL1 and DLX2 in the human telencephalon lies at the heart of a debate about the origins of interneurons in the human cerebral cortex (Molnar & Butt, 2013; Clowry, 2015). In rodents, GABAergic interneurons originate almost entirely outside the dorsal telencephalon in the ganglionic eminences and associated structures of the ventral telencephalon, such as the septum and preoptic area, from which they migrate tangentially into the cortex (De Carlos et al. 1996; Parnavelas, 2000; Marín & Rubenstein, 2003; Welagen & Anderson, 2011). Major studies in human and other primates have concluded that cortical interneurogenesis is essentially the same as in the rodent, occurring primarily in the ventral telencephalon, being extremely rare in the dorsal telencephalon where, if it does happen at all, it comes from ventral progenitor cells undergoing further divisions once they have migrated into the cortex (Hansen et al. 2013; Ma et al. 2013; Arshad et al. 2016) as happens in mouse in the later stages of corticoneurogenesis (Wu et al. 2011). Nevertheless, this contradicts a number of studies in the past 16 years that have provided evidence of GABAergic interneuron production within the primate cerebral cortex (Rakic & Zecevic, 2003; Petanjek et al. 2009; Jakovcevski et al. 2011; Zecevic et al. 2011; Radonjić et al. 2014a; Al‐Jaberi et al. 2015), and suggested that the increased proportion and sub‐specification of cortical interneurons in human arises from a degree of intracortical interneurogenesis, whether deriving from dorsal progenitors or ventral progenitors that have migrated dorsally and continued dividing (Radonjić et al. 2014a; Alzu'bi et al. 2017b).

This hypothesis originated from a highly cited study that suggested that as many as 65% of cortical interneurons are intrinsically generated (Letinic et al. 2002). This conclusion relied on the double expression of ASCL1 and DLX2 to define cells as of cortical origin, as Letinic et al. (2002) observed ASCL1 to be down‐regulated in migrating interneurons before they leave the MGE in organotypic cultures of human fetal forebrain. The reliability of ASCL1 as a marker for interneurons of cortical origin has been questioned on two grounds (Hansen et al. 2013). First, ASCL1 may be expressed by progenitors of glutamatergic neurons in the cortex and is not an exclusive marker for GABAergic neurons (Britz et al. 2006; Wilkinson et al. 2013). Secondly, interneurons originating from the CGE, now recognised to be a major source of interneurons, may continue to co‐express ASCL1 and DLX2 as they migrate into the cortex (Miyoshi et al. 2010).

To throw more light on this controversy, we have re‐examined the expression of ASCL1 and DLX2 in the human telencephalon from before formation of the cortical plate up to mid‐gestation. We have used double‐labelling of cells to learn more about the identity and state of differentiation of ASCL1 and DLX2 expressing cells in different locations and at different time points in development.

## Materials and methods

### Human tissue

Human fetal tissue from terminated pregnancies was obtained from the joint MRC/Wellcome Trust‐funded Human Developmental Biology Resource (HDBR, http://www.hdbr.org; Gerrelli et al. 2015). All tissue was collected with appropriate maternal consent and approval from the Newcastle and North Tyneside NHS Health Authority Joint Ethics Committee. Fetal samples ranging in age from 6.5 to 19 PCW were used. Ages were estimated from foot and heel to knee length measurements according to Hern (1984).

For immunostaining, brains were isolated and fixed for at least 24 h at 4 °C in 4% paraformaldehyde (Sigma‐Aldrich) dissolved in 0.1 m phosphate‐buffered saline (PBS). Once fixed, whole or half brains (divided sagittally) were dehydrated in a series of graded ethanols before embedding in paraffin. Brain samples were cut at 8‐μm section thickness in three different planes; horizontal, sagittal, and coronal, and mounted on slides.

### RNAseq

Full details of the origins, collection, preparation, sequencing and analysis of the human fetal RNA samples has been previously described (Ip et al. 2010; Lindsay et al. 2016; Harkin et al. 2017). Temporal lobes were removed and, in larger brains, divided into frontal and posterior sections; the remaining cortex was cut into coronal slices usually 5 mm wide containing both medial and lateral wall. The entire RNAseq dataset from which data were extracted for this study has been deposited at www.ebi.ac.uk/arrayexpress/experiments/E-MTAB-4840. High‐quality reads were then mapped to the human reference genome hg38 with Tophat2 (Kim et al. 2013). Reads aligned to genes and exons were counted with htseq‐count (Anders et al. 2014) and normalised RPKM calculated. Read length was 101 bp prior to trimming and 85 bp after trimming with no reads of < 20 bp retained. The minimum number of reads examined per sample was 63 million (average 90 million).

### Immunohistochemistry

This was carried out on paraffin sections according to previously described protocols (Harkin et al. 2016; Alzu'bi et al. 2017b). Antigen retrieval involved boiling in 10 mM citrate buffer pH6 for 10 min. Sections were incubated with primary antibody (diluted in 10% normal blocking serum in Tris‐buffered saline [TBS] pH 7.6) overnight at 4 °C. Details of primary antibodies are found in Table 1. Sections were incubated with biotinylated secondary antibody for 30 min at room temperature (Vector Laboratories Ltd., Peterborough, UK) 1 : 500 dilution in 10% normal serum in TBS followed by incubation with avidin‐peroxidase for 30 min (ABC‐HRP, Vector Labs) then developed with diaminobenzidine (DAB) solution (Vector Labs), washed, dehydrated, and mounted using DPX (Sigma‐Aldrich, Poole, UK). For double immunofluorescence, the Tyramide Signal Amplification (TSA) method was used, permitting double‐staining using same‐species antibodies. At the secondary antibody stage, sections were incubated with HRP‐conjugated secondary antibody for 30 min [ImmPRESS™ HRP IgG (Peroxidase) Polymer Detection Kit, Vector Labs) and then incubated in the dark for 10 min with fluorescein tyramide diluted at 1/500 (Tyramide Signal Amplification (TSA™) fluorescein plus system reagent (Perkin Elmer, Buckingham, UK) leaving fluorescent tags covalently bound to the section. Sections were then boiled in 10 mm citrate buffer pH6 to remove all antibodies and unbound fluorescein, incubated first in 10% normal serum then with the second primary antibody for 2 h at room temperature. Sections were again incubated with HRP‐conjugated secondary antibody followed by CY3 tyramide for 10 min (TSA™ CY3 plus system reagent, Perkin Elmer). Sections were dyed with 4′,6‐diamidino‐2‐phenylindole dihydrochloride (DAPI; Thermo Fisher Scientific, Cramlington, UK) and mounted using Vectashield Hardset Mounting Medium (Vector Labs). Extensive washing of sections was carried out between all incubations.

**Table 1:** Primary antibodies used in this study**.**

Primary antibody	Species	Dilution	Supplier	RRID Number (where available)
ASCL1	Mouse monoclonal	1/100	Santa Cruz, Heidelberg, Germany	AB_10918561
DLX2	Mouse monoclonal	1/200	Santa Cruz	Catalogue number SC‐393879
PAX6	Rabbit polyclonal	1/1500	Cambridge Bioscience, Cambridge, UK	AB_2565003
KI67	Mouse monoclonal	1/150	Dako, Ely, UK	AB_2142378
TBR1	Rabbit polyclonal	1/1000	Abcam, Cambridge, UK	AB_2200219
TBR2	Rabbit polyclonal	1/200	Abcam	AB_2142378
GAD67	Mouse monoclonal	1/1000	Merck Millipore, Watford, UK.	AB_2278725
NKX2.1	Mouse monoclonal	1/150	Dako	Not available
LHX6	Mouse monoclonal	1/200	Santa Cruz	AB_10649856
OLIG2	Rabbit polyclonal	1/1000	Merck Millipore	AB_10141047
COUP‐TFII	Mouse monoclonal	1/500	R&D Systems, Abingdon, UK.	AB_2155627
SOX2	Mouse monoclonal	1/500	Santa Cruz	AB_10842165

### Imaging and cell quantification

Images from immunoperoxidase‐stained sections were captured using a Leica slide scanner and Zeiss Axioplan 2 microscope; and from immunofluorescent‐stained sections with a Zeiss Axioimager Z2 apotome. Images were adjusted for brightness and sharpness using Adobe photoshop CS6 software. Cells were counted from five sections selected at intervals along the anterior–posterior axis of each fetal sample (12 PCW, *n* = 2 and 19 PCW, *n* = 2, therefore *n* = 10 for each age). Sections were observed under medium magnification; rectangular counting boxes 100 μm in width were placed over the ventricular/subventricular zones (VZ/SVZ) and intermediate zone/cortical plate (IZ/CP) delineated by the nuclear staining (DAPI).

## Results

### Expression of *ASCL1* and *DLX2* mRNA in cerebral cortex between 7.5 and 17 PCW

**Figure 1:**
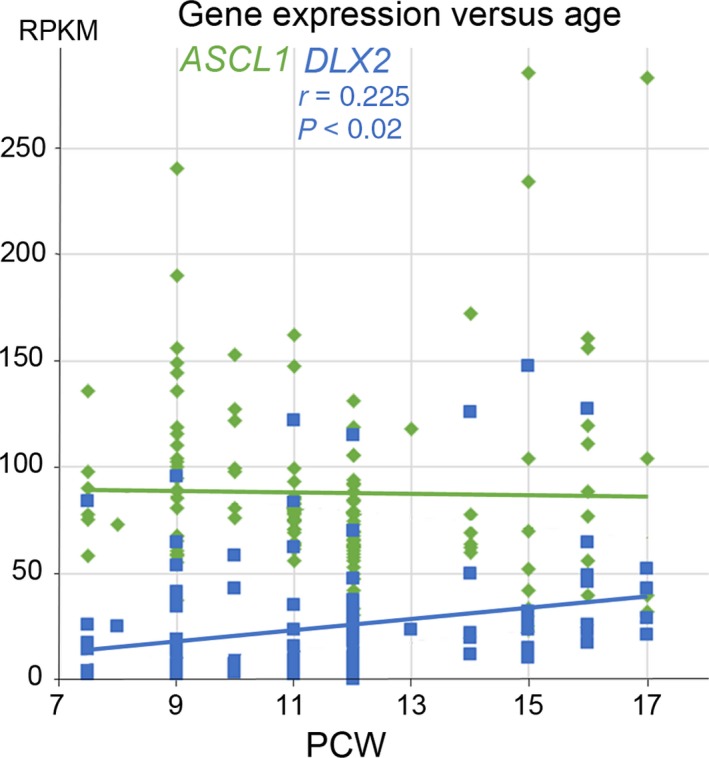
Change in *ASCL1* and *DLX2* expression with age. RNAseq data from a range of cortical samples from anterior to posterior locations taken between 7.5 and 17 post‐conceptional weeks (PCW). *ASCL1* showed high levels of expression maintained across the age range. *DLX2* showed an increasing level of expression with age which was statistically significant (*r* = correlation coefficient; P = probability of correlation not being real). RPKM; normalised reads per kilobase of transcript per million mapped reads.

A total of 130 samples of RNA were taken between 7.5 and 17 PCW from all regions of the cerebral cortex and subjected to quantitative RNAseq analysis (Fig. 1). We found that ASCL1 showed a high level of expression throughout the age range studied (mean 98 RPKM, first quartile of all protein coding genes; Harkin et al. 2017), whereas DLX2 expression increased significantly with age, from a moderate (~ 20 RPKM) to a high level (~ 40 RPKM) of expression, although not as high as ASCL1, on average (Fig. 1). For comparison, mean RPKM of typical reference genes *GAPDH* and *SDHA* was 2400 and 33.8 and for cortical transcription factors *EMX2* and *PAX6* was 162.5 and 87.6 over this age range, confirming that expression of transcription factors can be relatively high at this stage of development, including *ASCL1* and *DLX2*. Both genes showed a tendency towards higher expression from samples from the frontal and temporal cortex, but this was not statistically significant (not shown).

### Expression of ASCL1 and DLX2 protein in the early ventral telencephalon

**Figure 2:**
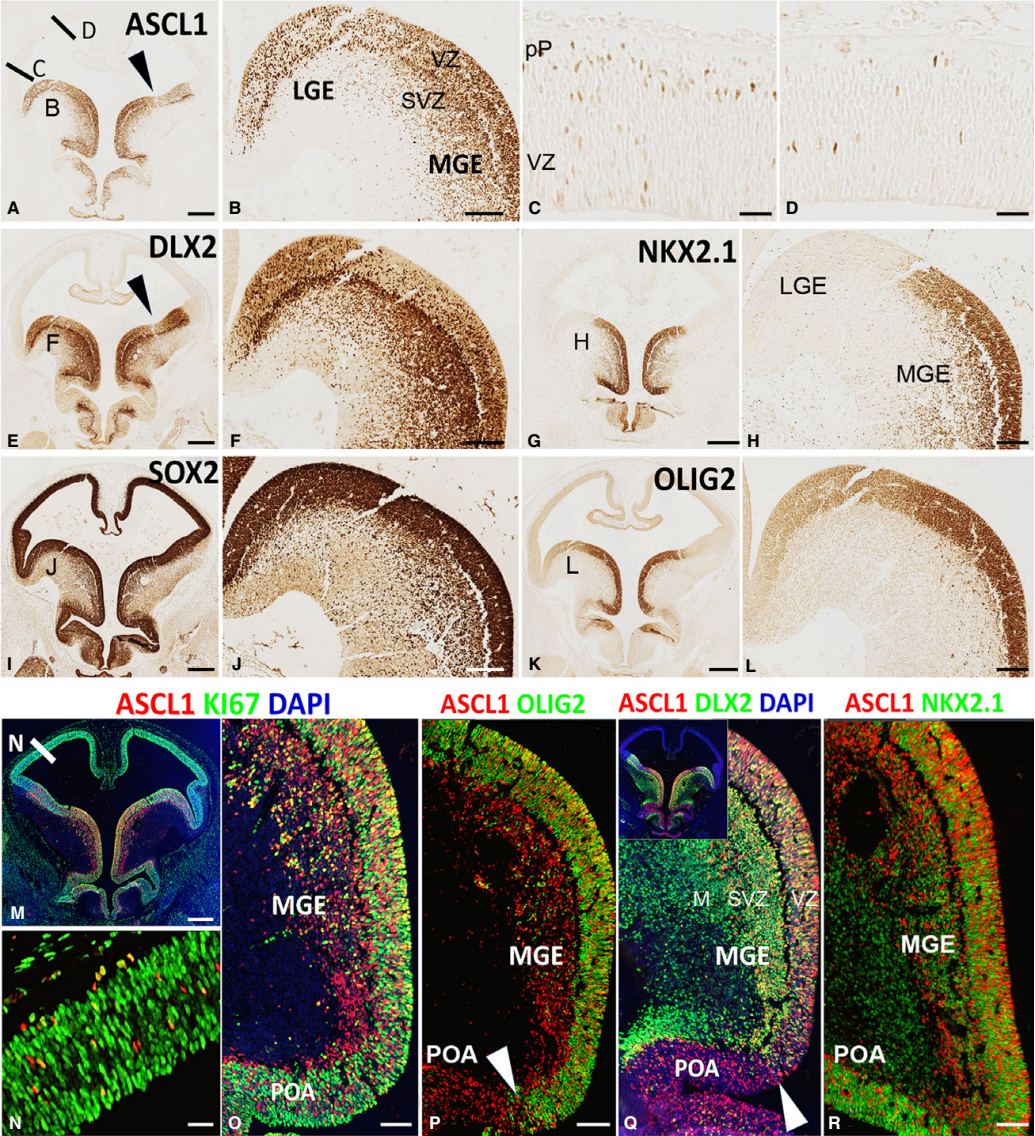
Expression of ASCL1, DLX2, and associated proteins at 6.5 PCW. (A) Shows high expression of ASCL1 in ganglionic eminences, preoptic area, and hypothalamus. Location of origin of panels (B,C,D) are marked. Arrowhead indicates boundary between LGE and MGE. (B) Shows ASCL1 expression was high in the ventricular zone (VZ) of both LGE and MGE and also high in the subventricular zone (SVZ) of the MGE. In the dorsal telencephalon expression was low, but higher laterally (C) than dorsally (D) with a few cells deep in the VZ but more present at the boundary of the VZ and the nascent post‐mitotic preplate (pP). (E) Shows high expression of DLX2 in ganglionic eminences, preoptic area, and hypothalamus with lower expression in the VZ of the ganglionic eminences and higher expression in the SVZ and overlying mantle. NKX2.1 expression was confined to MGE, preoptic area and parts of the hypothalamus (G,H). SOX2 expression marked all progenitor cells in the forebrain and illustrates the extent of the SVZ in the MGE (I,J) and strong expression of OLIG2 also delineated the MGE (K,L). Immunofluorescence double‐labelling showed that ASCL1^+^ cells of the telencephalon expressed cell division marker KI67 and thus were dividing neuroprogenitors (M–O). Co‐expression patterns of ASCL1 with OLIG2 (P) and DLX2 (Q) define the boundary between MGE and preoptic area (POA), although NKX2.1 is expressed in both regions (R). Scale bars: 500 μm (A,E,I,G,K,M), 100 μm (B,F,H,J,L,O,P,R for Q see P or R), 50 μm (C,D,N).

We examined the expression of ASCL1 and DLX2 in the forebrain at 6.5 PCW when the cortical preplate is barely formed but the ganglionic eminences are relatively well developed. Both transcription factors were densely expressed in the ventricular zone of the MGE and the LGE, although the boundary zone between the two was devoid of expression of either (Fig. 2A,B,E,F). The subventricular zone (SVZ) of the MGE was particularly expanded and densely populated with ASCL1^+^ and DLX2^+^ immunoreactive cells, in contrast to the LGE, which contained sparse numbers of ASCL1^+^ and DLX2^+^ cells in comparison. Both SOX2 (marker of neural stem cells) and NKX2.1 (marker of MGE‐derived precursor interneurons; Butt et al. 2008; Du et al. 2008) showed a pattern of expression similar to ASCL1 (Fig. 2G–J), indicating that ASCL1^+^ cells are mainly progenitors, which we confirmed by double‐labelling with cell division marker KI67 (Scholzen & Gerdes, 2000; Fig. 2M,N,O). OLIG2 is also a marker neural progenitor cells in the MGE (Miyoshi et al. 2007) and, unlike other MGE markers, its expression was mainly found in the ventricular zone (VZ) in cells that mostly co‐expressed ASCL1 (Fig. 2K,L,P). Only a few OLIG2^+^ cells were observed in the SVZ of the MGE.

Examination of ASCL1/DLX2 double‐labelling in the MGE showed that nearly all ASCL1^+^ cells expressed DLX2 (these cells are most likely progenitors; Fig. 2Q); however, the SVZ and mantle zone were also densely populated with cells expressing DLX2 only (these cells are most likely to be post‐mitotic; Fig. 2P). Thus, in the MGE, both ASCL1 and DLX2 were expressed in the progenitor cells, whereas postmitotic cells expressed only DLX2. However, in the preoptic area (POA), cells in the VZ and SVZ expressed either ASCL1 or NKX2.1 (Fig. 2R). No OLIG2 expression was seen in the POA (Fig. 2P), whereas DLX2 expression was limited to the SVZ (Fig. 2Q). Therefore, expression patterns of OLIG2 and DLX2 demarcate the boundary between the MGE and POA.

### Expression of ASCL1 protein in the cerebral cortex

ASCL1 immunoreactivity was also extensively expressed in the cerebral cortex as early as 6.5 PCW, being most highly expressed in the developmentally more advanced lateral cortical region (Fig. 2A,C) and with lower expression in the dorsal and medial regions (Fig. 2A,D). In all regions, ASCL1^+^ cells were mostly undergoing mitosis, co‐expressing KI67, suggesting they originate in the cortex (Fig. 2M,N). No DLX2 immunoreactivity was detected in the cortex at this stage of development (Fig. 2E).

**Figure 3:**
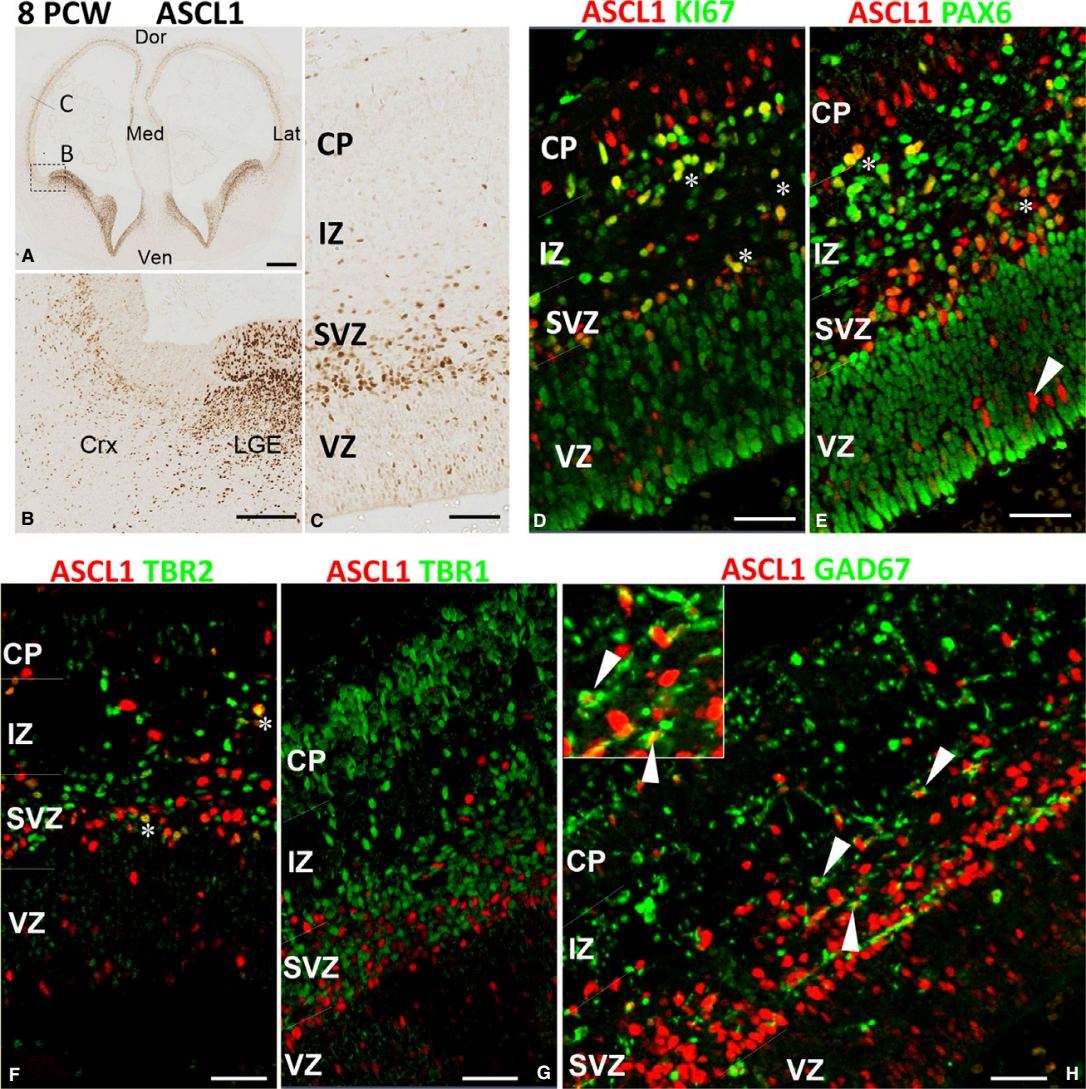
ASCL1 expression in the cortex at 8 PCW. (A) Anterior section of telencephalon; box shows the approximate location of panel (B) and the line of panel (C). Very high expression of ASCL1 in the ganglionic eminences and septum, and discernible expression in the cerebral cortex was observed (A,B). In the cortex, ASCL1 was predominantly expressed in subventricular zone (SVZ) and intermediate zone (IZ) with (D) many cells co‐expressing the marker for cell division KI67 (yellow, e.g. asterisks) and (E) the marker for radial glia, PAX6 (yellow, asterisks) in these locations. However, in the ventricular zone (VZ), co‐expression with either marker was rare (arrowhead). (F) ASCL1 was also co‐expressed with the marker of intermediate progenitor cells TBR2 (asterisks) but not with TBR1 (G) the marker for post‐mitotic neurons. (H) Co‐expression with GAD67, marker of GABAergic interneurons (arrowheads). Inset shows two examples of these double‐labelled cells at higher magnification. Dor, dorsal; Med, medial; Ven; ventral; Lat, lateral; CP; cortical plate. Scale bars: 500 μm (A), 100 μm (B), 50 μm (C–G) and 30 μm (H).

By 8 PCW, the stage of development at which the cortical SVZ emerges (Bayatti et al. 2008), the expression of ASCL1 continued in cells sparsely distributed within the VZ, but the majority of cortical ASCL1^+^ cells now resided in the SVZ, unlike in the adjacent LGE, where both VZ and SVZ were populated with ASCL1^+^ cells (Fig. 3A–C). We found that 60 ± 6% of ASCL1^+^ cortical cells were actively undergoing cell division (co‐expressing KI67; Fig. 3D). Furthermore, no gradient of ASCL1 expression was seen from the boundary of the GE to more remote regions of the cortex (Fig. 3). Together, these findings suggest that these ASCL1^+^ cells were likely to have originated in the cortex and had not migrated from the GE. Almost 45 ± 5% co‐expressed PAX6 (Fig. 3E; marker of cortical radial glial progenitors; Lui et al. 2011) and 38 ± 4% co‐expressed TBR2 (Fig. 3F; marker of cortical intermediate progenitors; Lui et al. 2011), suggesting that ASCL1 expression is in progenitors that typically give rise to glutamatergic pyramidal neurons, although no cells were observed that co‐expressed ASCL1 or the post‐mitotic glutamatergic neuron marker TBR1 (Fig. 3G).

**Figure 4:**
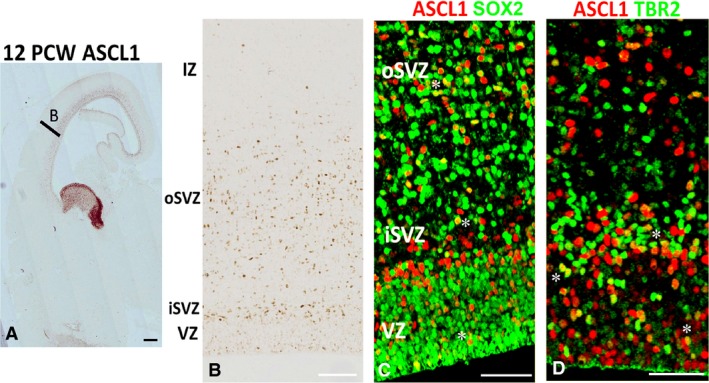
ASCL1 expression in the cortex at 12 PCW. (A) Section of telencephalon including MGE and LGE (high ASCL expression) with area B showing approximate location of panel (B). (B) Shows low expression of ASCL1 in the VZ but higher in the inner subventricular zone (iSVZ) and also throughout the outer subventricular zone (oSVZ). (C) Asterisks mark examples of double‐labelling of ASCL1 (red) with SOX2 (green, expressed by radial glia) and (D) mark examples of double‐labelling with TBR2 (expressed by intermediate progenitor cells). Scale bars:  1 mm (A), 100 μm (B) and 50 μm (C,D).

Notably, ASCL1/PAX6^+^ and ASCL1/TBR2^+^ cells were mainly found the SVZ, whereas the majority of ASCL1^+^ cells in the VZ were negative for PAX6 and TBR2 (Fig. 3E,F). They could have been either post‐mitotic cells in the process of downregulating PAX6 and/or TBR2; however, no cells expressing ASCL1 and the post‐mitotic marker TBR1 were seen in the VZ (Fig. 3G). Alternatively, they could be a distinct type of progenitor that gives rise to a population of cortical GABAergic interneurons in human cortex. In support of this idea we also observed many ASCL1^+^ cells that co‐expressed the GABAergic neuron marker GAD67 throughout the cortical wall (Fig. 3H) with 17% ± 3 of GAD67^+^ cells in the VZ/SVZ expressing ASCL1. By 12 PCW, expression of ASCL1 increased as the outer SVZ (oSVZ) compartment expanded (Fig. 4A,B). Most ASCL1^+^ cells in this compartment co‐expressed the radial glial cell markers SOX2 (Fig. 4C) or PAX6 (not shown) both in the SVZ but also more frequently in the VZ than at younger stages. ASCL1^+^/TBR2^+^ cells were largely confined to the boundary between the VZ and the inner SVZ (iSVZ) but were also observed in the VZ (Fig. 4D) Nevertheless, we continued to observe cells in the VZ that expressed ASCL1 only, and ASCL1^+^/GAD67^+^ cells throughout the cortical wall.

### MGE and septum provide the majority of interneuron precursors between 8 and 12 PCW

**Figure 5:**
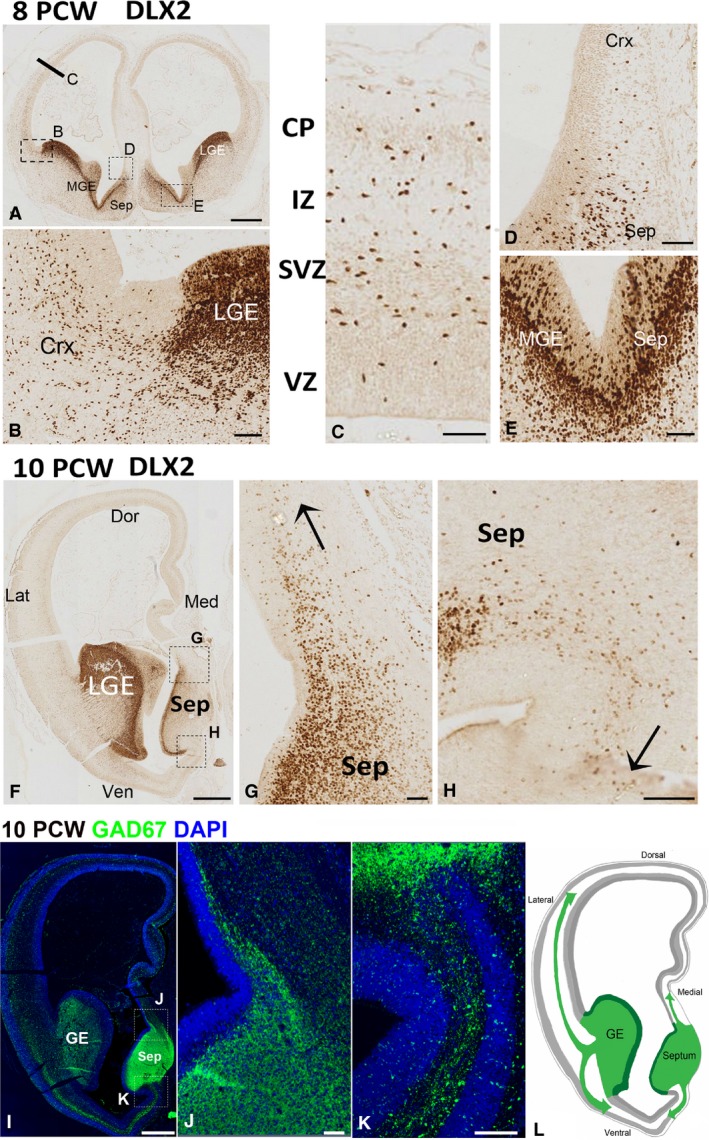
DLX2 expression 8–10 PCW. (A) DLX2 immunoreactivity in telencephalon at 8 PCW; bar and boxes show approximate location of panels (C,B,D). (B) Pallial/subpallial boundary. Dense expression of all layers of the lateral ganglionic eminence (LGE) with DLX2^+^ cells appearing to enter the cortex (Crx) principally through the SVZ. (C) Further dorsally in the cortical wall, DLX2^+^ cells are present in cortical plate (CP) intermediate zone (IZ) subventricular zone (SVZ) but with only a few in the ventricular zone (VZ). A few DLX2^+^ cells appear to migrate from the septum (Sep) to the dorsomedial cortex (D). Expression of DLX2 is continuous across the border between the medial ganglionic eminence (MGE) and septum, moderate in the ventricular layer and higher in the subventricular zone. (F) A more anterior section of the telencephalon at 10 PCW; high expression in the LGE, moderate in the septum. (G) Migration (arrow) of DLX2^+^ cells from septum to cortex dorsomedially. (H) Migration ventromedially. (I,J) Similar pattern of expression for GAD67, another marker of interneurons, that is highly expressed in the ganglionic eminences (GE) and septum (Sep), with GAD67^+^ cells appearing to migrate dorsally (J) and ventrally (K) from the septum. (L) A diagrammatic representation of the findings in (F–K) showing the lateral and medial pathways of migration of interneuron precursors from the subpallium to both dorsal and ventral cortex in the frontal lobe. Scale bars: 500 μm (A), 50 μm (B–E), 1 mm (F,I) and 100 μm (G,H,J,K).

From 8 PCW onwards, DLX2 immunoreactivity was observed to increase progressively in the cortical plate (Fig. 5A–C) in agreement with our RNAseq data (Fig. 1). These cells appeared to enter the cortex mainly from the ganglionic eminences (Fig. 5B) but a few DLX2^+^ cells also entered medial cortical wall from the septum (Fig. 5D). We have previously shown that the septum shares molecular features with the ganglionic eminences, and OLIG2^+^ cells from the septum can migrate into the medial wall of the anterior cortex in human (Alzu'bi et al. 2017b). Here we show that the proliferative zone (VZ and SVZ) of human septum, similar to the medial ganglionic eminence, is also expanded markedly in size and cell number starting from 8 PCW (Fig. 5E). The number of DLX2^+^ cells entering the cortex from septum considerably increased by 10 PCW (Fig. 5F–H). In addition, DLX2^+^ interneuron migration from septum was not only restricted in dorsal direction to reach the medial cortical wall (Fig. 5G) but also exhibited a ventral pathway to populate the ventral part of anterior cortical regions (Fig. 5H). GAD67 immunofluorescence exhibited a similar pattern of expression (Fig. 5I–J), with expression in the ganglionic eminences but also intense immunoreactivity in the septum in the frontal lobe (Fig. 5I). GAD67^+^ cells also appeared to migrate dorsally (Fig. 5J).

**Figure 6:**
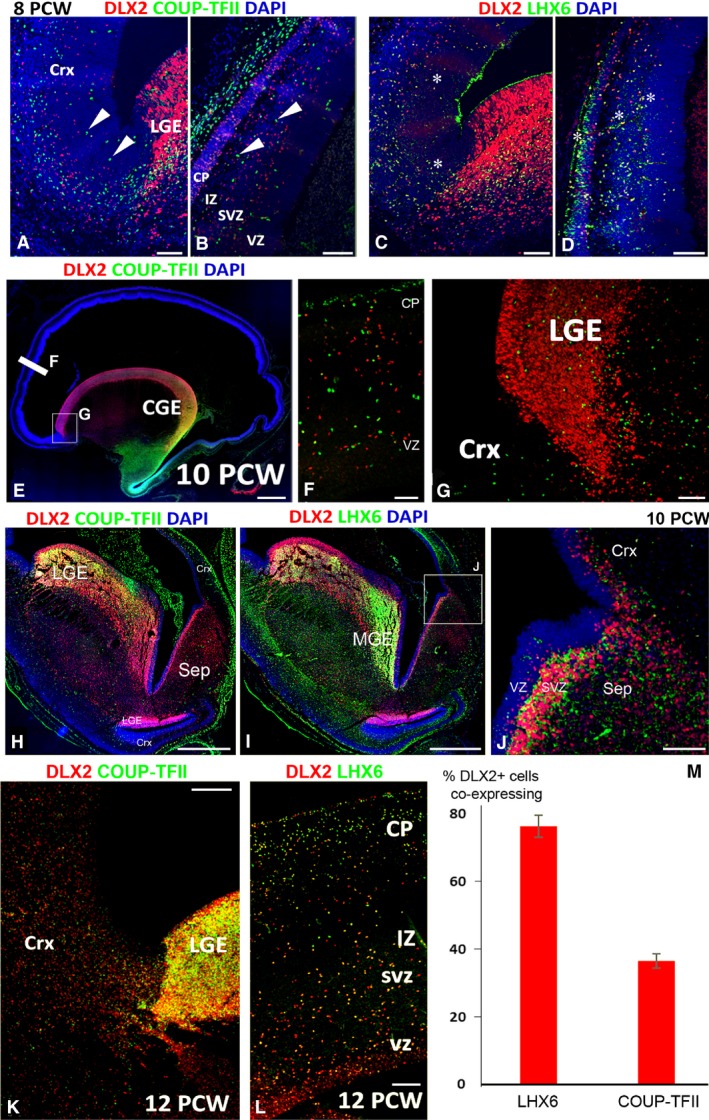
Co‐expression of DLX2 with LHX6 and COUP‐TFII. (A) At 8 PCW, both DLX2^+^ cells (red) and COUP‐TFII^+^ cells (green) can be seen in the cortex (Crx) but the populations are non‐overlapping. Arrowheads show examples of COUP‐TFII^+^/DLX2^–^ cells throughout the cortical wall (A,B). However, extensive co‐expression of DLX2 and COUP‐TFII (yellow, asterisks) was observed (C,D). The situation was similar at 10 PCW (E) with extensive co‐expression in the ventral caudal ganglionic eminence (CGE, yellow). No double‐labelled cells were present in the cortex or in the LGE (F,G). DLX2^+^/COUP‐TFII^+^ co‐expression (yellow) was observed in the LGE at the boundary with the lateral cortex but not at the LGE/ventral cortex boundary, or in the MGE or septum (H). However, DLX2 was co‐localised with LHX6 in the MGE (I) and also to a lesser extent in septum (J). By 12 PCW, the numbers of COUP‐TFII cells in the LGE had greatly increased and double‐labelled cells were present throughout the cortical wall (yellow, K,L). Nevertheless, cell counts demonstrated that a higher proportion of DLX2^+^ cells co‐expressed LHX6 than COUP‐TFII (M). LGE, lateral ganglionic eminence; CP, cortical plate; IZ, intermediate zone; SVZ, subventricular zone; VZ, ventricular zone. In (J) error bars represent the standard error of the mean. Scale bars: 100 μm (A–D,G,L), 1 mm (E), 50 μm (F) and 500 μm (H,I,K).

As DLX2 is considered a universal marker for all GABAergic interneuron precursors (Panganiban & Rubenstein, 2002) we utilised this to explore the relative contribution of the MGE and CGE to the cortical interneuron population at an early stage of cortical development (8‐12 PCW). Double‐labelling of DLX2^+^ cells for either COUP‐TFII (marker of CGE‐derived interneurons; Kanatani et al. 2008; Alzu'bi et al. 2017b) or LHX6 (marker of MGE‐derived interneurons; Liodis et al. 2007) was attempted. Very few DLX2^+^ cells co‐expressed COUP‐TFII in the cortex at 8 PCW. In addition, most of COUP‐TFII^+^ cells found in the cortex at this stage were negative for DLX2 (Fig. 6A,B). These cells either originated from the cortex itself, where COUPTFII^+^ progenitor cells giving rise to glutamatergic neurons can be found in ventral and temporal cortex (Alzu'bi et al. 2017a,b), or perhaps from MGE/LGE boundary, a neurogenic domain showing high COUP‐TFII expression (Alzu'bi et al. 2017b) but few DLX2^+^ cells (Fig. 2E). In contrast to COUP‐TFII, the majority of DLX2^+^ cells in the LGE (the main corridor for tangential migration of MGE‐derived cells into the cortex) and cortical wall were LHX6^+^ (Fig. 6C,D). Similarly, at 10 PCW, DLX2^+^/COUP‐TFII^+^ cells were very rare in the cortex (Fig. 6E,F). DLX2^+^/COUP‐TFII^+^ cells were mainly seen the CGE but not in LGE and cortex (Fig. 6E–G). Patterns of gene expression in the septum closely resembled the MGE, with little expression of COUP‐TFII, some expression of DLX2 in the VZ, and expression of LHX6 in the SVZ, although there was less LHX6 expression in the septum compared with MGE (Fig. 6H–J).

At 12 PCW, the number of DLX2/COUP‐TFII^+^ cells notably increased in the LGE (Fig. 6K), suggesting a prominent anterior migration of CGE‐derived interneuron precursors through LGE at this stage, although numbers of double‐labelled cells in the cortex remained low (Fig. 6K). However, plentiful DLX2^+^/LHX6^+^ cells were observed in the cortical wall (Fig. 6L) and quantification of the number of DLX2/COUP‐TFII^+^ cells and DLX2/LHX6^+^ cells in the cortical wall at 12 PCW showed that 76 ± 3% of DLX2^+^ cells co‐expressed LHX6 and only 37 ± 2% of DLX2^+^ cells expressed COUP‐TFII (Fig. 6M). In summary, at the early stage of development, similar to rodent, the MGE is the major source of cortical interneurons in human.

### DLX2 marks new populations of interneurons and interneuron precursors by mid‐gestation

**Figure 7:**
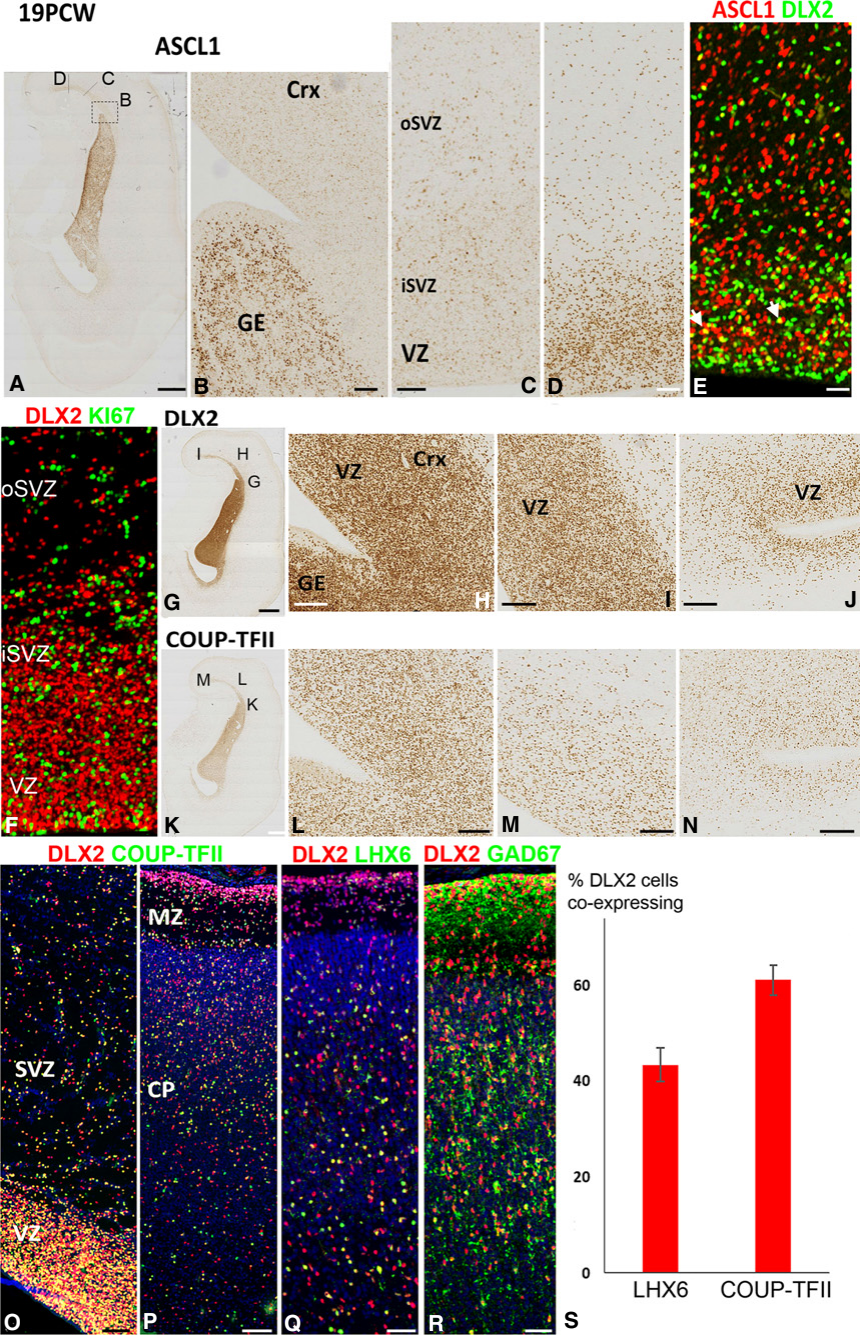
ASCL1 and DLX2 expression at 19 PCW. (A) Coronal section of cortex and ganglionic eminence immunostained for ASCL1 showing approximate location of panels (B,C,D). (B) High expression in the ganglionic eminence (GE) and very low expression in the lateral cortex which increases at more dorsomedial cortical locations (C,D) in both the inner and outer subventricular zone (iSVZ, oSVZ), but especially in the ventricular zone (VZ). (E) Co‐expression of ASCL1 (red) and DLX2 (green) was rare but observed in a few cells (yellow, e.g. arrowheads). (F) However, no co‐expression of DLX2 (red) with KI67 (green) was observed in the cortex. DLX2 expression was highest in the GE and in the lateral cortex close to the pallial/subpallial border, particularly in the VZ (G,H). More dorsomedially, the concentration of DLX2^+^ cells decreased (I,J). COUP‐TFII exhibited a similar pattern of expression (K–N), and DLX2 (red) and COUP‐TFII (green) were widely co‐expressed (yellow) throughout the cortical wall (O,P). A proportion of DLX2 cells also co‐expressed LHX6 (yellow, Q) and extensively co‐expressed GAD67 (R). At this stage of development, a higher proportion of DLX2^+^ cells co‐expressed COUP‐TFII than LHX6 (S). Scale bars: 2 mm (A,G,K), 200 μm (B), 25 μm (C–E for F see E) 200 μm (L,M,N) and 50 μm (O–R).

By 19 PCW, we observed a marked change in the localisation of both ASCL1 and DLX2 protein expression. Expression of ASCL1 remained low in the ventricular zone of the ventral pallium close to the pallial/sub‐pallial boundary (PSB; Fig. 7A–C) but increased progressively in locations in the dorsal pallium away from this zone (Fig. 7A,D) exceeding expression in the SVZ. Some co‐expression with DLX2 was also seen (Fig. 7E), although ASCL1^+^ and DLX2^+^ cell populations did not extensively overlap. DLX2 expression was also markedly different at this stage of development. DLX2 expression increased greatly in the cortical VZ, although there was little, if any, co‐expression with KI67 (Fig. 7F) demonstrating that DLX2 was not expressed by a population of progenitor cells. This expression in the cortical VZ was in a counter‐gradient to the ASCL1 expression observed in the VZ at this stage, with high expression at the PSB (Fig. 7G, H) decreasing towards more dorsomedial locations (Fig. 7I,J). No double‐labelling of DLX2 with PAX6 was observed (not shown) but there was an increase in immunoreactivity for COUP‐TFII in the VZ of the dorsal cortex that mirrored the increase in DLX2 expression (Fig. 7K–N), being higher at the PSB and lower dorsomedially. We observed extensive co‐expression of DLX2 with COUP‐TFII throughout the cortical wall but particularly in the VZ and SVZ (Fig. 7O,P). DLX2 was still co‐expressed with LHX6 in the cortical plate but only in subset of cells (Fig. 7Q). Practically all DLX2^+^ cells co‐expressed GAD67 in the MZ and CP (Fig. 7R). There was a high density of DLX2^+^ cells in the subpial layer of the MZ, but only a small proportion of cells co‐expressed COUP‐TFII or LHX6 (Fig. 7O–Q). By this stage of development a higher proportion of putative DLX2^+^ interneurons co‐expressed COUP‐TFII than LHX6 (Fig. 7S).

## Discussion

This study has confirmed that two transcription factors, ASCL1 and DLX2, which play central roles in the specification of forebrain GABAergic neurons, including cortical interneurons, are highly expressed in the dorsal as well as ventral telencephalon in the early development of the human cerebral cortex. ASCL1 expression is high from the beginning of cortical plate development and mostly localised to a population of dividing cells in the inner subventricular zone (iSVZ). However, these cells co‐express PAX6 or TBR2 and would be predicted to give rise to glutamatergic neurons. Towards mid‐gestation, expression increases in the ventricular zone. DLX2 expression progressively increases in cells that appear to be migrating from the ganglionic eminences and septum via lateral and medial routes at various levels in the cortical wall. Initially, these cells predominantly co‐express LHX6, deriving from both the MGE and septum. By mid‐gestation, a later wave of DLX2/COUP‐TFII^+^ cells predominates, many of which are located in the cortical ventricular zone. However, we found no evidence that these cells derived from cortical progenitor cells, but instead the cells choose to migrate through this region, presumably having predominantly derived from the ventral CGE (Alzu'bi et al. 2017b) and entering the cortex directly from the ventral CGE into the temporal cortex, or into other cortical regions via the LGE.

### ASCL1‐expressing cortical progenitor cells

Although it was originally reported that ASCL1 expression is primarily expressed in the progenitor zones of the ventral telencephalon in rodents (Guillemot & Joyner, 1993; Porteus et al. 1994), more recent studies have revealed that ASCL1 is expressed at low levels in progenitors of the dorsal telencephalon (Britz et al. 2006), where it is proposed to have different functions to the ventral telencephalon. Instead of maintaining proliferation and inducing GABAergic identity (Casarosa et al. 1999; Yun et al. 2002) it is proposed to maintain proliferation (Britz et al. 2006; Castro et al. 2011) and promote the translocation of intermediate progenitor cells (IPCs) to the iSVZ (Britz et al. 2006). Experiments in rodents have demonstrated oscillating expression of Neurog1/2 and Ascl1 in progenitors, with a switch to stable expression of Neurog1/2 only leading to exit from the cell cycle and differentiation into cortical deep layer glutamatergic neurons (Wilkinson et al. 2013).

We have observed high levels of *ASCL1* expression in developing human cerebral cortex and ASCL1^+^ cells to be predominantly in the iSVZ, but also in the oSVZ where they co‐expressed PAX6 or TBR2, transcription factors marking cells that are part of the cortical glutamatergic lineage (Hevner et al. 2006). In rodents, ectopic expression of ASCL1 in NEUROG2‐expressing cortical progenitors results in their translocation from the VZ to the boundary of the SVZ and VZ (Britz et al. 2006). This suggests that elevated ASCL1^+^ in human cortical progenitors may promote translocation from the VZ to the SVZ while antagonising the effect of NEUROG1/2 sufficiently to prevent downregulation of progenitor cell markers such as PAX6 and TBR2, thus maintaining a larger pool of dividing cells in the SVZ, contributing to the evolutionary expansion of cortical neuron numbers seen in human compared with rodents (Lui et al. 2011). Interrogation of single cell transcriptome data (https://bioinf.eva.mpg.de/shiny/sample-apps/ShinyCortex/; Kageyama et al. 2018) revealed that in human cortex *ASCL1* is more highly expressed in cells of SVZ than VZ origin (Pollen et al. 2015) and in cells identified as basal progenitors (IPCs and basal radial glia of the oSVZ) over apical radial glia of the VZ and newborn neurons (Camp et al. 2015); However, higher expression of *ASCL1* is not characteristic of mouse cortical IPCs (Telley et al. 2016), providing more evidence that a stable population of ASCL1 expressing IPCs and bRG may be an evolutionary feature of primate development.

We found no evidence that ASCL1‐expressing cortical IPCs could provide a substantial source of cortically derived interneurons in primates as has been suggested (Letinic et al. 2002; Petanjek et al. 2009). Over 80% expressed either of the pro‐glutamatergic transcription factors PAX6 and TBR2, whereas none co‐expressed ASCL1 and DLX2, as was observed in the ventral telencephalon, except at 19 PCW, where a few such cells were present. Nevertheless, small numbers of ASCL1^+^ cells were observed in the VZ which failed to express PAX6/TBR2, and other cells were observed that co‐expressed ASCL1 and GAD67, the GABAergic neuron marker, as previously reported at mid‐gestation (Jakovcevski et al. 2011). Therefore, it remains a possibility that a small number of ASCL1^+^ progenitors may give rise to GABAergic neurons, particularly at later stages, where it has been reported that mRNA levels of *ASCL1* increase dramatically between 16 and 19 PCW along with GSX2, a transcription factor important in the differentiation of calretinin‐positive interneurons of CGE origin (Radonjić et al. 2014b). Our RNAseq study only extended as far as 17 PCW but showed no trend towards a dramatic increase in *ASCL1* expression towards mid‐gestation, although we did see a large increase in ASCL1 immunoreactive cells in the VZ in parts of the cortex at 19 PCW. It has been reported that interneuron precursors of CGE origin maintain expression of ASCL1 (Miyoshi et al. 2010). However, we saw the largest increase in expression away from the boundary with the CGE, with only a few cells co‐expressing DLX2 and ASCL1. Therefore, it is more likely that ASCL1/GAD67^+^ cells may be of cortical rather than CGE origin.

It has also been suggested that in rodent, ASCL1 expression drives the differentiation of a subset of Cajal–Retzius cells in the ventral and medial pallium (Dixit et al. 2011). We found relatively higher expression of ASCL1 in the ventral pallium than in other cortical areas, but very low expression in the medial pallium and cortical hem. Therefore, the extent to which ASCL1 plays a role in human Caja–Retzius cell development is not clear.

### DLX2‐expressing interneuron precursors

Between 8 and 12 PCW, we observed increasing levels of expression of *DLX2* and increasing numbers of DLX2^+^ cells throughout the cortical wall, not confined to proliferative zones and with a decreasing gradient away from the ganglionic eminences, which is agreement with previous studies (Hansen et al. 2013; Arshad et al. 2016) that concluded that DLX2 is expressed by post‐mitotic interneuron precursors that do not express markers of cell division and maintain expression as they migrate to the cortex, as has been shown in mouse (Cobos et al. 2006). However, by 19 PCW, an increasing proportion of DLX2^+^ cells were present in the VZ, and bearing in mind that most reports of cortical interneuron production have examined mid‐gestational tissue (Zecevic et al. 2011; Radonjić et al. 2014a) we explored this further. We found no evidence that these cells were undergoing division and we noticed that their density was highest in regions close to the CGE. Therefore, it is unlikely that these DLX2^+^ cells in the VZ represent a population of cortically derived interneuron progenitors appearing around 19 PCW. Instead, it may be that as the population of progenitor cells in the VZ becomes depleted, this lamina of the cortical wall becomes a preferred route for the migration of interneuron precursors into the cortex.

This study confirmed the existence and importance of a medial migration pathway from septum to the medial cortical in human forebrain development reported previously from observations of OLIG2 expression (Alzu'bi et al. 2017b). Although minimal at 8 PCW, from 10 PCW onwards, large numbers of DLX2^+^ cells appeared to streaming dorsomedially and ventromedially into the cortex (Fig. 5L) from the septum, with patterns of expression of ASCL1, DLX2 and LHX6 similar to that seen in the MGE. Evidence from rodent studies suggest that no interneurons derive from the septum (Rubin et al. 2010) and all interneuron precursors from the ganglionic eminences enter the cortex via lateral pathways ((Pleasure et al. 2000; Wonders & Anderson, 2006; Morozov et al. 2009). It would appear that, in human, medial pathways are much more prominent than in rodent, perhaps to help provide the higher relative numbers of interneurons present in the primate compared with rodent cortex. It is worth noting that a medial migratory pathway for Nkx2.1^+^ precursors from the MGE to the medial pallium has recently been reported in the shark (Quintana‐Urzainqui et al. 2015) and therefore is not evolutionarily novel to the human forebrain and may be present, but overlooked, in the rodent.

We also asked to what extent DLX2^+^ cells originated from the MGE or the CGE, depending upon the co‐expression of characteristic transcription factors LHX6 (Liodis et al. 2007) and COUP‐TFII (Kanatani et al. 2008), respectively. We found that the majority of DLX2^+^ cells co‐expressed LHX6 in the earlier stages of development, with a relatively larger proportion of cells expressing COUP‐TFII at later stages. Interneurons of MGE origin are distributed relatively evenly between layers in rodent and monkey cortex, whereas those of CGE origin tend to populate the superficial layers (Ma et al. 2013). It therefore makes sense that COUP‐TFII^+^ interneuron precursors should, in the main, arrive later in development when the superficial cortical layers are being established, especially as, in rodents at least, the CGE produces interneurons destined for all layers of the cortex simultaneously (Miyoshi et al. 2010).

It is also worth noting that we observed many cells in the subpial granular layer of the marginal zone which co‐expressed GAD67 and DLX2 at 19 PCW. This confirms previous observations that this provides a migratory route for substantial numbers of interneuron precursors at later stages of development of the primate brain (Rakic & Zecevic, 2003).

## Conclusion

Although ASCL1 and DLX2 play important roles in specification and differentiation of GABAergic neurons of the forebrain, this study shows that their extensive expression in the human dorsal telencephalon does not provide much evidence in support of the hypothesis that in human there is significant intracortical generation of inhibitory interneurons. However, we suggest that ASCL1 expression may be elevated, relative to rodents, in a population of glutamatergic neuron progenitor cells that are chiefly found within the inner subventricular zone. This may play a role in maintaining progenitor cell status and in expanding the number of neurons produced. The extended expression of DLX2 in migrating interneuron precursors revealed the importance of medial migration pathways for cells of an MGE‐like phenotype from septum to cortex, which has not been reported in rodent.

## Conflict of interest

The authors have no conflict of interest to declare.
